# Induction of Apoptosis Coupled to Endoplasmic Reticulum Stress through Regulation of CHOP and JNK in Bone Marrow Mesenchymal Stem Cells from Patients with Systemic Lupus Erythematosus

**DOI:** 10.1155/2015/183738

**Published:** 2015-05-19

**Authors:** Genkai Guo, Yan Meng, Wei Tan, Yunfei Xia, Chun Cheng, Xiaolan Chen, Zhifeng Gu

**Affiliations:** ^1^Department of Rheumatology, Affiliated Hospital of Nantong University, Nantong 226001, China; ^2^Jiangsu Province Key Laboratory for Inflammation and Molecular Drug Target, Medical College, Nantong University, Nantong 226001, China; ^3^Department of Nephrology, Affiliated Hospital of Nantong University, Nantong 226001, China

## Abstract

Previous studies indicated that bone marrow mesenchymal stem cells (BM-MSCs) from patients with systemic lupus erythematosus (SLE) exhibited the phenomenon of apoptosis. In this study, we aimed to investigate whether apoptosis of BM-MSCs from SLE patients were dysregulated. In this paper, endoplasmic reticulum stress (ERS) was evidenced by increased expression of phosphorylated protein kinase RNA-like ER kinase (PERK) and inositol-requiring protein-1 (IRE-1). We also found the activation of downstream target eukaryotic translation initiator factor 2*α* (eIF 2*α*) and CCAAT/enhancer-binding protein- (C/EBP-) homologous protein (CHOP) in BM-MSCs from SLE patients. Interestingly, we discovered that 4-phenylbutyric acid (4-PBA), a selective inhibitor of ERS, blocked the apoptosis of BM-MSCs from SLE patients and alleviated the level of Jun N-terminal kinase1/2 (JNK1/2) and CHOP. Furthermore, blockage of PERK signaling expression by siRNA not only significantly reduced the expression of CHOP, but also activated the anti-apoptotic regulator B-cell lymphoma-2 (Bcl-2). Blockage of IRE-1 or JNK1/2 by siRNA resulted in the decreased expression of JNK1/2 and proapoptosis protein Bcl-2 associated protein X (BAX). These results implicated that ERS-mediated apoptosis was a critical determinant of BM-MSCs from SLE patients.

## 1. Introduction

Systemic lupus erythematosus (SLE) is an autoimmune inflammatory disease characterized by multiorgan involvements and various clinical manifestations [1]. Current treatments have achieved successes, but some patients with refractory SLE are still suffering from poor prognosis. Allogenic mesenchymal stem cells transplantation (MSCT) has shown to be a powerful strategy in the treatment of refractory SLE patients [2, 3]. However, syngeneic bone marrow MSC transplantation (BM-MSCT) was ineffective [4, 5]. These studies suggested that bone marrow mesenchymal stem cells (BM-MSCs) might be somewhat functionally deficient and participated in the pathological process of SLE. Previous studies found that hematopoietic stem cells (HSC) from SLE patients were prone to apoptosis with defects in their function [6]. The paper of Li et al. showed that there were increased frequencies of apoptosis and aging in BM-MSCs from SLE patients in comparison with control groups [7]. Our data revealed that BM-MSCs from SLE patients exhibited some abnormalities of the cytoskeleton and ultrastructure [8–11]. All these studies suggested that researches in the apoptosis of BM-MSCs from SLE had a profound and historical significance in the evolution process of the disease. In this study, we aimed to discover the molecular mechanism contributing to the pathogenesis of the disease.

Protein folding in the endoplasmic reticulum (ER) is impaired under various physical and pathological conditions, termed endoplasmic reticulum stress (ERS). ERS is buffered by the activation of the unfolded protein response (UPR), a homeostatic signaling network that orchestrates the recovery of ER function, and failure to adapt to ERS results in apoptosis [12, 13]. The UPR signals through three distinct stress sensors located at the ER membrane: protein kinase RNA-like ER kinase (PERK), inositol-requiring protein-1 (IRE-1), and activating transcription factor-6 (ATF-6) [14]. Among them, PERK, whose intrinsic kinase activity is induced by oligomerization, regulates the phosphorylation of the eukaryotic translation initiation factor 2*α* (eIF 2*α*), which induces the suppression of global mRNA translation to protect cells against ERS [15]. Previous studies showed that the transcription factor CCAAT/enhancer-binding protein- (C/EBP-) homologous protein (CHOP) operates at the ERS pathways to introduce the apoptosis of cells [16, 17]. Over expression of CHOP plays an important role in apoptosis [18, 19]. IRE-1-mediated activation of Jun N-terminal kinase (JNK) contributes to cell death [20]. These results suggested that the induction of ERS was closely related with apoptosis. However, it was still not confirmed whether ERS was involved in the pathological process of BM-MSCs from SLE patients.

In the present paper, we observed dilated, distorted, and swollen ER in apoptosis BM-MSCs from SLE patients and upregulated expression of phosphorylated IRE-1 and PERK. We then found that, in BM-MSCs from SLE patients, 4-phenylbutyric acid (4-PBA), the inhibitor of ERS [21], could partly reduce the apoptosis situation. Further, ERS suppressed the expression of B-cell lymphoma 2 (Bcl-2) via introduction of CHOP and led to the activation of Bcl-2 associated protein X (BAX) through activation of JNK, which contributed to ERS-induced apoptosis in BM-MSCs from SLE patients.

## 2. Methods

### 2.1. Recruitment of SLE Patients

Twelve SLE patients between 16 and 43 years of age (mean 27.3 ± 7.81 years) were enrolled in this study (Table 1). Our previous study had indicated that the BM-MSCs from treated- and untreated-SLE patients were senescent [8], so we did not group in this study. The SLE diagnosis was made based on the criteria proposed by the American College of Rheumatology [22]. The Systemic Lupus Erythematosus Disease Activity Index (SLEDAI) was used to measure the disease activity. All patients were categorized as active using a cutoff SLEDAI score of eight. Ten healthy subjects were used as the normal group (Table 1). All research subjects were females with a similar age distributions (mean 26.8 ± 7.45 years). All participators gave consent to the study, which was approved by the Ethics Committee of the Affiliated Hospital of Nantong University.

### 2.2. Isolation, Cell Culture, and Identification of BM-MSCs from SLE and Normal Subjects

BM-MSCs were generated from bone marrow aspirate according to a technique described previously [7]. Five milliliters of heparinized BM were mixed with an equal volume of phosphate-buffered saline (PBS). Then, the resuspended cells were layered over Ficoll solution (1.077 g/mL) and centrifuged at 2,000 ×g for 25 min at room temperature. The mononuclear cells were collected at the interface and resuspended in low-glucose Dulbecco-Modified Eagle Medium (L-DMEM) supplemented with 10% heat inactivated fetal bovine serum (FBS). Then, the cells were plated at a density of 2 × 10^7^ cells per 25 cm^2^ dish and cultured at 37°C in a 5% CO_2_ incubator. The medium was replaced, and nonadherent cells were removed after 5 days and every 3 days thereafter. When the BM-MSCs became nearly confluent, the adherent cells were released from the dishes with 0.25% trypsin–EDTA (Gibco, USA) and were then replated at a density of 1 × 10^6^ cells per 25 cm^2^ dish. Flow cytometric analysis showed that the cells were positive for CD29, CD44, CD105, and CD166 but negative for CD14, CD34, CD38, CD45, and HLA-DR. After 3 passages (p3), cells were used for the following studies. After 4 passages (p4), the cells were treated with or without 4-phenylbutyric acid (4-PBA) (P21005) (100 *μ*M) for 24 hours. Transfection of cells with various mammalian expression conducted by Lipofectamine 2000 (Invitrogen, Carlsbad, CA, USA) was in accordance with the methods provided by manufacturer's specification.

### 2.3. FCM Analysis for Cell Apoptosis

The Annexin V-FITC/PI Apoptosis Detection Kit (Becton Dickinson, USA) was employed to evaluate the apoptosis of P4 BM-MSCs from 12 SLE patients and 12 normal controls by using FCM analysis. After being washed with iced cold PBS, 1 × 10^5^cells were resuspended in 100 *µ*L of binding buffer. Five microliter Annexin V and 5 *µ*L propidium iodide (PI) were added to the cells. After incubation for 15 min (25°C) in the dark, 400 *μ*L of 1×binding buffer was added to each tube and FCM analysis was performed immediately. Data acquisition and analysis were assayed by BD LSRII analyzer (Becton Dickinson, USA) using “CellQuest” software. Annexin V (+) and PI (−)/PI (+) cells were considered as apoptotic cells.

### 2.4. Transmission Electron Microscopic Examination

Electron microscopy was performed by using the methods described as previous [23]. Briefly, BM-MSCs were washed twice with PBS and fixed in 4% glutaraldehyde. They were then postfixed with 1% OsO4, dehydrated stepwise in increasing concentrations of ethanol, and embedded in Epon 812 epoxy resin. Ultrathin sections were then localized and viewed with a transmission electron microscope (JEM-1230, JEOL Ltd., Japan).

### 2.5. Western Blot Analysis

After being subjected to the indicated treatments, approximate 5 × 10^6^ BM-MSCs were washed with cold PBS three times and lysed with cell lysis solution. Sample buffer was added to cytosolic extracts, and after boiling for 10 min, equal amounts of supernatant from each sample were fractionated by 10% SDS-polyacrylamide gel electrophoresis (SDS-PAGE) and electro transferred to polyvinylidenedifluoride (PVDF) membranes (Millipore, USA). The membranes were blocked with 5% skim milk for 2 h at room temperature. Then the membranes were incubated with antibody for p-PERK, PERK, p-IRE1, IRE1, BAX, Bcl-2 (1 : 800; Cell Signaling), caspase-3, eIF 2*α*, CHOP, p-JNK1/2, JNK1/2, p-extracellular signal-regulated kinase (ERK), ERK, p-p38, p38 (1 : 800; Santa Cruz) at 4°C overnight. At last, immunoreactive bands were then detected by incubation with conjugates of anti-rabbit IgG with horseradish peroxidase (1 : 2,000; Southern-Biotech) for 2 h and visualized by using enhanced chemiluminescence system (ECL; Pierce Company, USA).

### 2.6. Immunofluorescent Staining

BM-MSCs grown on coverslips were fixed with 4% paraformaldehyde at 4°C for 40 min, followed by several rinses in phosphate-buffered saline (PBS). Nonspecific binding sites were blocked at room temperature for 2 h with 5% normal horse serum (Sigma-Aldrich, St. Louis, MO, USA) or normal donkey serum (Sigma-Aldrich, St. Louis, MO, USA) diluted in 0.1% Triton X-100-PBS. BM-MSCs were then incubated overnight at 4°C with primary antibodies: CHOP (Cell signaling, MA, USA), JNK1/2 and caspase-3 (green; Santa Cruz, CA, USA) at 1 : 300 dilution with blocking buffer, followed by a mixture of fluorescein isothiocyanate and tetramethylrhodamineisothiocyanate-conjugated secondary antibodies (BD Biosciences) for 2 h at 4°C. BM-MSCs were finally incubated with DAPI (blue; Sigma, MO, USA), which was used to stain the nucleus for 30 min at 37°C. The slides were visualized by using a fluorescent microscope (Leica, Microsystems, Germany). All photomicrographs shown in this study were representative of multiple experiments.

### 2.7. Transfection with siRNA

CHOP and IRE-1 siRNA were designed by siGENOME ON-TARGET plus SMARTpool siRNA purchased from Dhamarcon RNAi Technologies. CHOP (DDIT3) target sequences are GGUAUGAGGACCUGCAAGA, CACCAAGCAUGAACAAUUG, GGAAACAGAGUGGUCAUUC, CAGCUGAGUCAUUGCCUUU. IRE-1 (ERN1) target sequences are CUACCCAAACAUCGGGAAA, CUCCAGAGAUGCUGAGCGA, AUAAUGAAGGCCUGACGAA, GUCCAGCUGUUGCGAGAAU. Nontargeting control sequences were not provided. JNK1/2 and PERK siRNA (1, # 6232, 2, # 6233) was purchased from Cell Signaling Technology, Inc. (Danvers, MA) and sequences were not provided. Cells at 50 to 60% confluence were transfect with siRNA (20–50 nM) using the RNAifect Transfection Regaent (QIAGEN) according to the manufacturer's protocol. Cells were cultured for 48 h, and then treated with vehicle for an additional 48 h. Proteins were then isolated for western blotting.

### 2.8. Statistical Analysis

Statistical analysis was performed by one-way ANOVA. The intergroup comparisons (post-hoc analysis) among the data with equal variances were performed with the least significant difference (LSD) method, while Tamhane's T2 method was used for the data with unequal variances. *P* < 0.05 was considered to be significant.

## 3. Results

### 3.1. Apoptosis of BMSCs from SLE Patients

**Figure 1:**
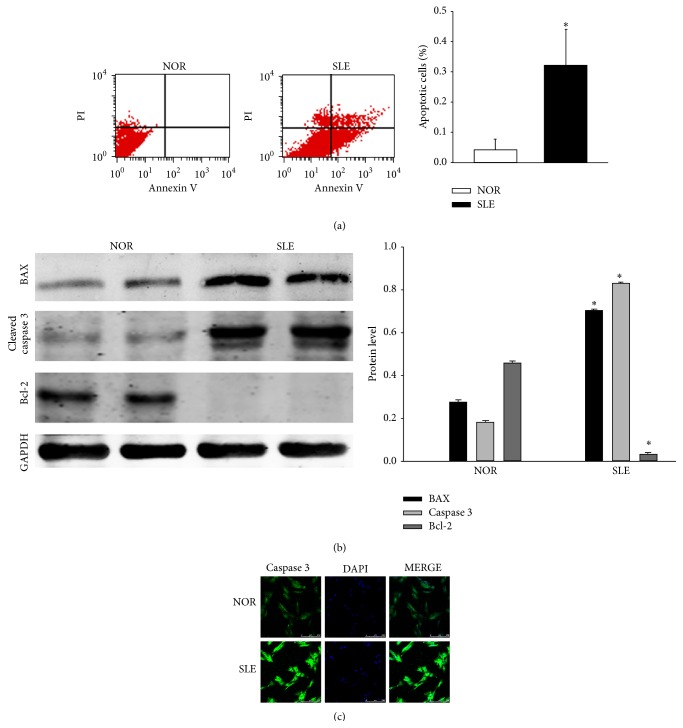
Apoptosis of BMSCs from SLE patients. (a) BM-MSCs at passage 4 were isolated and harvested from SLE patients and normal controls, and analyzed by Annexin V-FITC/PI staining. Annexin V-positive cells were 4.1 ± 3.7% in normal controls, 32.3 ± 12.0% in SLE patients (*P* < 0.05). (b) Western blot analysis of BAX, active caspase-3 and Bcl-2 was performed in BM-MSCs from normal controls and SLE patients. (c) BM-MSCs from normal controls and SLE patients were fixed and stained with antiactive caspase-3. FITC-labeled secondary antibody was used (green fluorescence) Nuclei were stained with DAPI (blue fluorescence). Images were captured by a confocal laser microscope. (Bar represents mean ± SD, ^*^ *P* < 0.05 compared with the normal group).

The primary culture of BM-MSCs was successful in 12 cases of SLE patients and 10 cases of healthy donors. We sought to assess apoptotic cells using FCM analysis by Annexin V-FITC/PI staining at the fourth passage. A significant increase was discovered in Annexin V-positive cells among BM-MSCs from SLE patients (32.3 ± 12.0%) compared to normal controls (4.1 ± 3.7%) (*P* < 0.05) (Figure 1(a)). To investigate the functions of intrinsic pathway during the apoptosis of BM-MSCs from SLE patients, the expressions of Bcl-2 and BAX were measured by western blot. Results revealed that the expression of Bcl-2 significantly decreased in BM-MSCs from SLE patients compared to normal controls. The level of BAX and cleaved caspase-3 significantly increased in BM-MSCs from SLE patients compared to normal controls (Figure 1(b)). We also discovered that the immunoreactivity of caspase-3 was enhanced in BM-MSCs from SLE patients (Figure 1(c)). All these results showed that BM-MSCs were apoptotic in SLE patients group.

### 3.2. ERS Was Involved with BM-MSCs from SLE Patients

**Figure 2:**
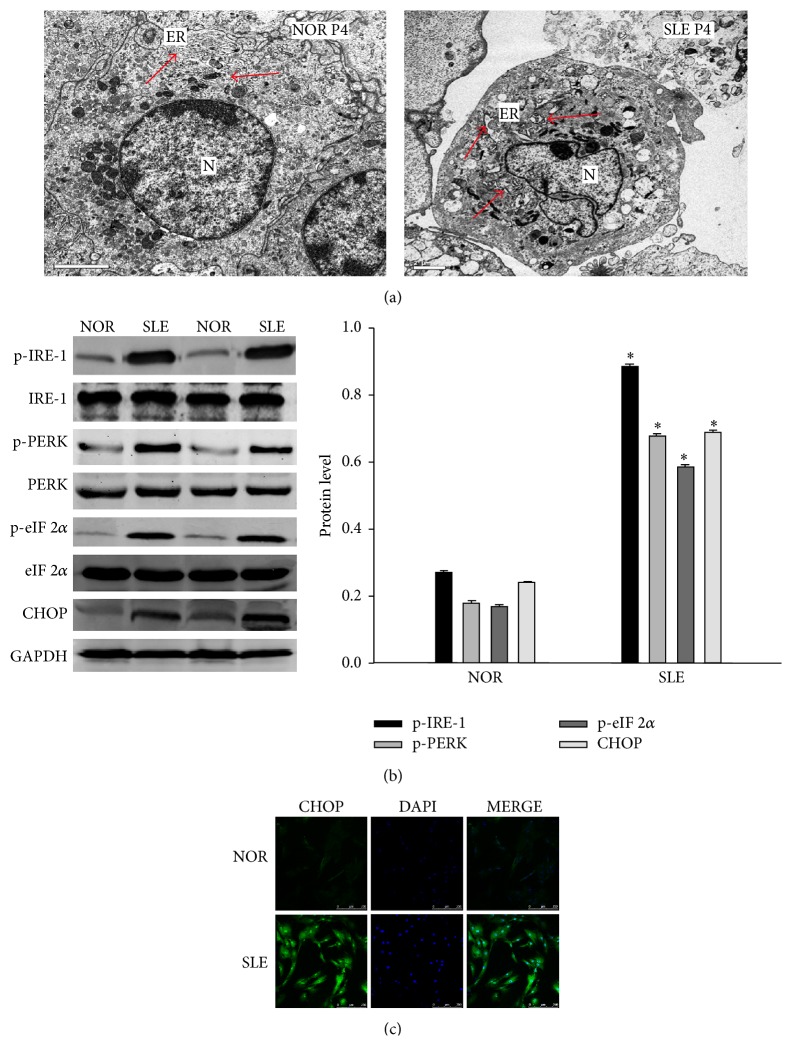
ERS was involved with BM-MSCs from SLE patients. (a) ER ultrastructural changes in the BM-MSCs from SLE patients and normal controls. The BM-MSCs were processed and examined under a transmission electron microscope. Compared with the control, there was an extensive increase in the size of the ER in BM-MSCs from SLE patients, in which arrows indicate protein aggregates within ER lumen. ER, endoplasmic reticulum; N, nucleus. Arrow indicates the normal ER or the dilatation of ER. (b) Upregulated expression of genes involved in ERS in BM-MSCs of SLE patients and normal controls. The levels of p-IRE-1, p-PERK, p-eIF 2*α* and CHOP were analyzed by using Western Blot analysis. Total IRE-1, PERK, eIF 2*α* and GAPDH were used as control for protein loading. (c) Immunofluorescence analysis of CHOP expression in the control group or SLE group. (Bar represents mean ± SD, ^*^ *P* < 0.05 compared with the normal group).

Electron microscopy was used to evaluate the ultrastructural changes of ER in the BM-MSCs. Cells in the control appeared to be normal with relatively healthy-looking ER, mitochondria and nuclei. Dilated and distorted ER, swollen mitochondria, and condensation of chromatin were found in the BM-MSCs from SLE patients; more protein aggregates were seen within the ER lumen than that observed in control groups, indicating severe ERS in BM-MSCs from SLE patients. In addition, we also found a mixed feature of apoptosis in BM-MSCs from SLE patients, such as shrunken nuclei, disrupted cell membranes, damaged organelles and formation of numerous vacuoles in the cytoplasm (Figure 2(a)). Upregulation of ERS transducer phosphorylated IRE-1 and PERK were observed but not ATF-6. The similar results were found in western blot analysis that eIF 2*α* and CHOP protein expression were significantly upregulated in BM-MSCs from SLE patients, respectively (Figure 2(b)). To study the involvement of CHOP in the apoptosis of BM-MSCs from SLE patients, we first investigated whether the translocation of CHOP protein turned into the nucleus. CHOP nuclear translocation represented the carrying of stress signal into the nucleus and therefore a functional activation. In BM-MSCs from SLE patients, CHOP was apparently more abundant in the nucleus than in that of controls (Figure 2(c)). These results suggest that ER stress accompanied apoptotic BM-MSCs from SLE patients.

### 3.3. Intervention to ERS Partly Rescued the Apoptosis of BM-MSCs from SLE Patients

**Figure 3:**
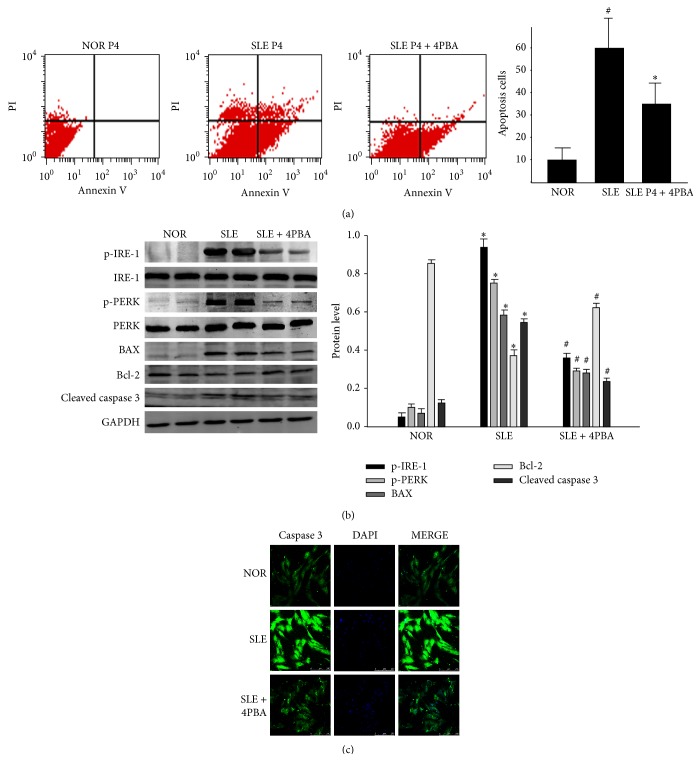
Intervention to ERS can partly rescue the apoptosis of BM-MSCs from SLE patients. BM-MSCs of SLE patients and normal controls were treated with (+) or without (−) 4-PBA (100 *μ*M) from the same time point for the indicated time. (a) Cell viability was assessed by FCM analysis. (b) Cell lysates were analyzed for the levels of p-IRE-1, p-PERK, BAX, Bcl-2 and cleaved caspase-3 via western blot. (Bar represents mean ± SD, ^*^ *P* < 0.05 compared with the normal group, ^#^ *P* < 0.05 compared with the SLE group). (c) Immunofluorescence analysis was used to analyze the level of cleaved caspase-3.

To determine whether ERS was involved with the apoptosis in BM-MSCs from SLE patients, we treated BM-MSCs from SLE patients with 100 *μ*M 4-PBA as the previous study described [24]. Interestingly, when treated with 4-PBA, the frequency of apoptotic cells measured by FCM analysis decreased distinctly in SLE group (Figure 3(a)). The relatively lower level of phosphorylated IRE-1 and PERK in 4-PBA-treated cells indicated the alleviation of ERS in BM-MSCs from SLE patients. Additionally, the relative level of Bcl-2 could be partly restored in 4-PBA-treated BM-MSCs from SLE patients, and the expression of BAX and cleaved caspase-3 was lower than that in BM-MSCs from SLE patients (Figure 3(b)). We also discovered the same result with the immunofluorescence analysis of cleaved caspase-3 (Figure 3(c)). Taken together, these data suggested that ERS played an important role in the apoptosis of BM-MSCs from SLE patients.

### 3.4. The Role of ERS in Apoptosis of BM-MSCs from SLE Patients

**Figure 4:**
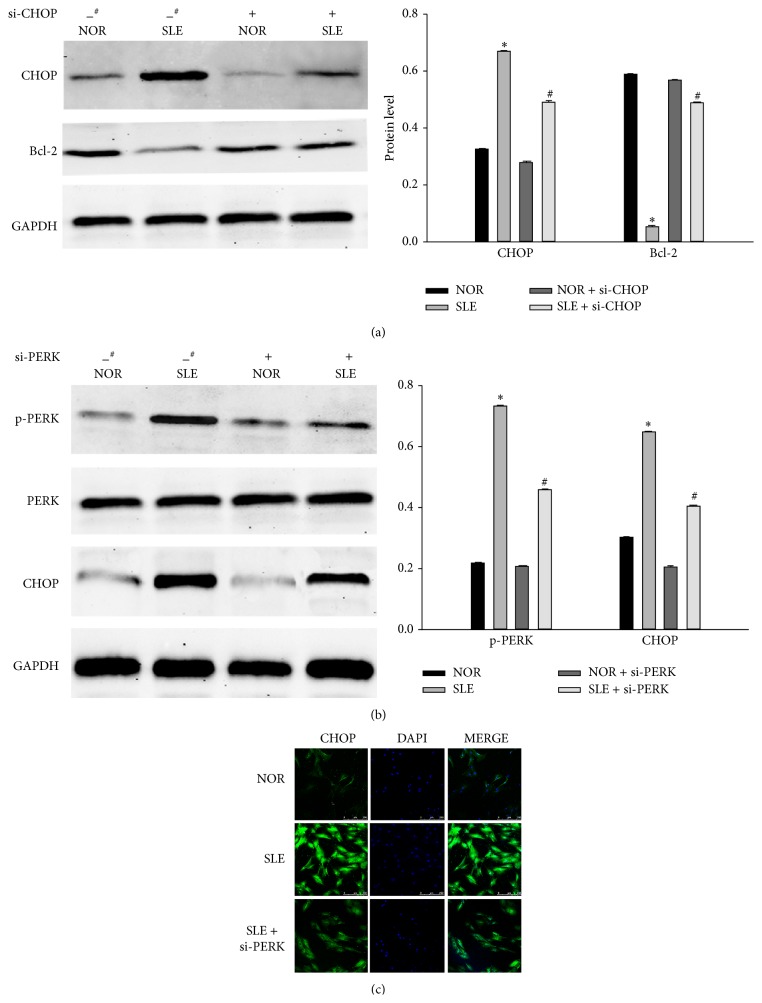
The role of ERS in apoptosis of BM-MSCs from SLE patients. (a) BM-MSCs of SLE patients and normal controls were transfected with scramble siRNA (^#^) and 40 nM CHOP siRNA respectively, for 48 h using the RNAifect transfection reagent. Western blot analysis was performed for CHOP and Bcl-2 respectively. (Bar represents mean ± SD, ^*^ *P* < 0.05 compared with the normal group, ^#^ *P* < 0.05 compared with the SLE group). (b) BM-MSCs of SLE patients and normal controls were transfected with scramble siRNA (^#^) and 50 nM PERK siRNA, respectively, for 48 h using the RNAifect transfection reagent. Western blot analysis was used for PERK and CHOP respectively. GAPDH was used as an internal control. (Bar represents mean ± SD, ^*^ *P* < 0.05 compared with the normal group, ^#^ *P* < 0.05 compared with the SLE group). (c) Immunofluorescence analysis was performed for CHOP expression.

We further used a siRNA approach to determine the role of ERS in the apoptosis of BM-MSCs from SLE patients. Cells were transfected with 40 nM siRNAs for CHOP and PERK for 48 h, respectively. Western blot analysis demonstrated that transfection of si-CHOP resulted in a suppression of CHOP expression induced by ERS in BM-MSCs from SLE patients, as compared with cells transfected with control scrambled siRNA. Bcl-2 increased in BM-MSCs from SLE patients transfected with CHOP siRNA as compared to scrambled siRNA-transfected cells (Figure 4(a)). Since CHOP gene promoter contained binding sites for PERK induced by ERS, we further characterized the role of PERK in ERS-induced cell death. We silenced PERK expression by 50 nM si-PERK transfection for 48 h. PERK siRNA significantly blocked phosphorylated PERK protein expression induced by ERS. In addition, CHOP was also suppressed by PERK siRNA transfection (Figure 4(b)). The immunofluorescence of CHOP was decreased by si-PERK transfection (Figure 4(c)). Taken together, these results indicated that ERS-induced apoptosis might be involved in the apoptosis of BM-MSCs from SLE patients, at least in part, through the upregulation of PERK and CHOP proteins expression.

### 3.5. The Role of MAPK on ERS-Induced Apoptosis of BM-MSCs from SLE Patients

**Figure 5:**
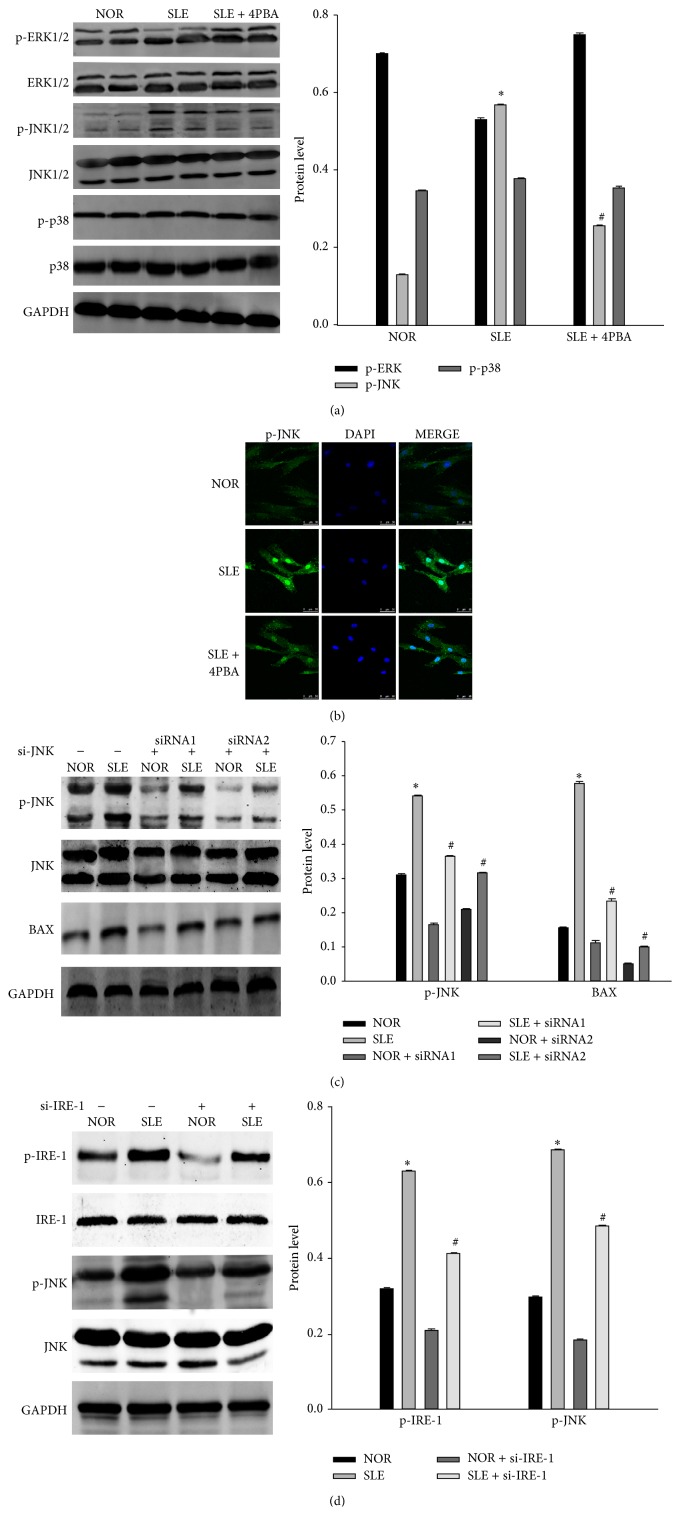
The role of MAPK on ERS-induced apoptosis of BM-MSCs from SLE patients. (a) BM-MSCs of SLE patients and normal controls were treated with (+) or without (−) 4-PBA (100 *μ*M) from the same time point for the indicated time. The levels of p-ERK1/2, total ERK1/2, p-JNK1/2, total JNK1/2, p-p38, and total p38 were detected by western blotting respectively. (b) Immunofluorescence analysis of p-JNK1/2 expression in the control group and SLE group treated with or without 4-PBA. (c) BM-MSCs of SLE patients and normal controls were transfected with scramble (^#^) or 20 nM JNK1/2 siRNA for 48 h using the RNAifect transfection reagent. Western blot analysis was performed for p-JNK1/2 and BAX. (d) BM-MSCs of SLE patients and normal controls were transfected with scramble (^#^) or 50 nM IRE-1siRNA for 48 h using the RNAifect transfection reagent. Western blot analysis was performed for p-JNK1/2 and p-IRE-1. (Bar represents mean ± SD, ^*^ *P* < 0.05 compared with the normal group, ^#^ *P* < 0.05 compared with the SLE group).

Activation of mitogen-activated protein kinase (MAPK) was implicated in the regulation of ERS-induced apoptosis. We investigated the condition of MAPK in BM-MSCs from SLE patients. Phosphorylation of JNK 1/2 and ERK 1/2 were detected in BM-MSCs from SLE patients, but not p38 MAPK. The ERK 1/2 phosphorylation downregulation occurred in BM-MSCs from SLE patients, which could be restored in 4-PBA-treated group. On the contrary, the phosphorylation of JNK1/2 increased in BM-MSCs from SLE patients and was partly reduced with 4-PBA-treated (Figure 5(a)). After the treatment with 4-PBA, we observed, by immunofluorescence analysis, that the high expression of phosphorylated JNK 1/2 in BM-MSCs from SLE was reversed, especially in the nucleus (Figure 5(b)). These findings suggested that JNK1/2 activation was involved in ERS introduced apoptosis of BM-MSCs from SLE patients. To further characterize the role of JNK 1/2 in the apoptosis of BM-MSCs from SLE patients, we silenced JNK 1/2 expression by specific siRNA transfection. JNK 1/2 expression and phosphorylation were reduced by si-JNK transfection. In addition, the ERS-induced expression of BAX was also reduced by si-JNK transfection (Figure 5(c)). Then we silenced IRE-1 expression by si-IRE-1 transfection. IRE-1 protein expression induced by ERS was significantly blocked by IRE-1 siRNA. In addition, JNK1/2 was also inhibited by IRE-1 siRNA transfection (Figure 5(d)). These findings suggested that JNK1/2 activated by IRE-1 was involved in ERS-induced apoptosis in BM-MSCs from SLE patients.

## 4. Discussion

Recently, SLE has been postulated by some to be a stem cell disorder disease [7, 25]. Our studies demonstrated that BM-MSCs from SLE patients appeared to show abnormity in early passages, in accordance with a previous report [8, 9]. Li et al. showed that there were increased frequencies of apoptosis in BM-MSCs from SLE patients in comparison with control groups [7]. Thus, we speculated that the apoptosis might be involved with functional abnormalities in BM-MSCs from SLE patients, which in turn participated in the pathogenesis of SLE. Our results revealed the involvement of ERS in the apoptosis of BM-MSCs from SLE patients. Activation of the UPR plays a protective role to cells under ERS [26]. Physiological processes that demand a high rate of protein synthesis and secretion must sustain activation of the UPR's adaptive programs without triggering cell death pathways. However, prolonged activation of UPR by excessive ERS can convert its role to cytotoxic by activation of multiple apoptotic pathways in mammalian cells [27]. The mechanisms initiating apoptosis under conditions of irreversible ER damage are now partially understood and may involve a series of complementary pathways [28]. The ERS transducer proteins ATF-6, IRE-1 and PERK constitute the core stress regulator of the UPR, and transducer signals from the ER to the cytoplasm and nucleus after ERS [29–31]. Previous papers reported that the induction of ERS had closely relationship with apoptosis [32, 33]. Chronic ER stress leads to BAX-dependent apoptosis through the transcriptional upregulation of BCL-2 homology 3 (BH3-) only proteins, such as BCL-2-interacting mediator of cell death (BIM) and p53 upregulated modulator of apoptosis (PUMA), which are upstream BCL-2 family members [34]. The transcription of one of the key UPR pro-apoptotic players, termed CHOP, is positively controlled by the PERK–ATF4 axis [28]. CHOP promotes both the transcription of BIM and the down regulation of BCL-2 expression, contributing to the induction of apoptosis [28, 34]. In addition to CHOP, ATF4 and p53 are also involved in the direct transcriptional upregulation of BH3-only proteins under ER stress [34]. Many other complementary mechanisms are proposed to induce cell death under excessive ER stress, including activation of the BH3-only protein BH3-interacting domain death agonist (BID) by caspase 2, as well as ER calcium release, which may sensitize mitochondria to activate apoptosis [28, 35]. Under certain conditions, IRE1*α* activation is also linked to apoptosis, possibly through its ability to activate MAPKs and the subsequent downstream engagement of the BCL-2 family members, as well as the degradation of mRNAs encoding for key folding mediators through CHOP [36]. In this study, we discovered swollen ER in apoptotic BM-MSCs from SLE patients. The activation of IRE-1 and PERK significantly increased in the apoptotic BM-MSCs from SLE patients. Interestingly, the intervention to ERS could recover the phenomenon of apoptosis in BM-MSCs from SLE patients. This suggested that ERS played an important role in the process of apoptosis in BM-MSCs from SLE patients.

One of the characteristic features of ERS was the increased expression of CHOP. It was a member of the C/EBP family of transcription factors. Although CHOP was originally identified as part of the DNA damage response pathway, its induction was probably the most sensitive to ERS conditions where it played a key role in the ERS-induced apoptosis, through the mechanisms of that are not entirely delineated. The PERK-eIF2*α*-ATF4 arm of the UPR is required to induce CHOP protein expression [17]. CHOP-induced cell death has been associated with downregulation of BCL-2 levels [19]. In this study, we found that the expression of CHOP increased in BM-MSCs from SLE patients but decreased when treated with 4-PBA. Cells were transfected with siRNA for CHOP. Western blot analysis demonstrated that transfection of si-CHOP resulted in a suppression of CHOP expression induced by ERS in BM-MSCs from SLE patients, as compared with cells transfected with control scrambled siRNA. Bcl-2 increased in BM-MSCs from SLE patients transfected with CHOP siRNA as compared to scrambled siRNA-transfected cells. Since CHOP gene promoter contained binding sites for PERK induced by ERS, we further characterized the role of PERK in the ERS-induced apoptosis. We silenced PERK expression by si-PERK transfection. PERK siRNA significantly blocked PERK protein expression induced by ERS. In addition, CHOP was also suppressed by PERK siRNA transfection. These results indicated that ERS-induced cell death through the upregulation of PERK and CHOP proteins expression.

The greater MAPK signaling cascade consists of a sequence of successively acting kinases that result in the dual phosphorylation and activation of terminal kinases p38, JNKs, and extracellular signal-regulated kinases (ERKs) [37]. Furthermore, activation of MAPKs has been implicated in the regulation of gene expression in the ERS signaling cascade and is involved in many aspects of the control of cellular proliferation and apoptosis. The JNK pathway has been shown to be a positive regulator of ERS induced apoptosis [38]. ERS do so indirectly through the activation of downstream molecules JNK1/2, which regulate the expression and activity of various pro- and antiapoptotic proteins such as BCL-2 family members and further push the cell down the path of apoptosis [39]. Previous reports showed activation of p38 and JNK were responsible for oxidative stress induced apoptosis [32]. But their respective roles in apoptosis of BM-MSCs from SLE patients were unknown. In our study, phosphorylation of JNK1/2 was observed in BM-MSCs from SLE patients. Increasing and sustaining expression of IRE-1 and its downstream target JNK1/2 were reduced when treated with 4-PBA in BM-MSCs from SLE patients. Inhibition of IRE-1 expression by siRNA led to downregulation of JNK1/2, and partially reduced the expression of proapoptotic protein BAX. These findings implicated that ERS mediated the apoptosis via the cooperation with the JNK pathway.

## 5. Conclusions

In conclusion, we provided evidence that ERS participated in the process of apoptosis in BM-MSCs from SLE patients. Our study also found that ERS induced the apoptosis through upregulating expression of JNK1/2 and CHOP. All of these findings suggested that SLE BM-MSCs were involved with ERS, which might implicate the possible involvement of BM-MSCs in the pathogenesis of SLE. These findings would suggest a novel strategy for the potential therapies of SLE patients.

## Figures and Tables

**Table 1:** Details of 10 controls and 12 SLE patients.

Control/ patient	Age	Disease duration	Current treated	SLEDAI
P1	16	1 year	Pred 20 mg/day HCQ 0.2/day	11
P2	24	8 months	Pred 15 mg/day HCQ 0.2/day LEF 0.2/day	14
P3	19	2 years	Pred 15 mg/day HCQ 0.2/day LEF 0.2/day	9
P4	37	1 year	Pred 17.5 mg/day HCQ 0.2/day	13
P5	22	16 months	Pred 12.5 mg/day HCQ 0.2/day CTX 0.4/2 weeks	16
P6	32	2 years	None	16
P7	33	3 years	Pred 15 mg/day HCQ 0.2/day	11
P8	41	5 years	Pred 7.5 mg/day HCQ 0.2/day	8
P9	18	3 days	None	9
P10	27	2 days	Pred 12.5 mg/day HCQ 0.2/day	12
P11	28	2 days	None	14
P12	31	4 days	None	21
C1	18			
C2	31			
C3	21			
C4	25			
C5	24			
C6	22			
C7	37			
C8	43			
C9	25			
C10	22			

Pred: prednisolone; HCQ: hydroxychloroquine.

LEF: leflunomide; CTX: cyclophosphamide.

## Abbreviations

SLE: Systemic lupus erythematosus
MSC: Mesenchymal stem cells transplantation
BM-MSCT: Bone marrow MSC transplantation
BM-MSCs: Bone marrow mesenchymal stem cells
HSC: Hematopoietic stem cells
ER: Endoplasmic reticulum
ERS: Endoplasmic reticulum stress
UPR: Unfolded protein response
IRE-1: Inositol-requiring protein-1
PERK: Protein kinase RNA-like ER kinase
ATF-6: Activating transcription factor-6
eIF 2*α*: Eukaryotic translation initiation factor 2*α*
CHOP: CCAAT/enhancer-binding protein (C/EBP)-homologous protein
JNK: Jun N-terminal kinase
BAX: Associated protein X
Bcl-2: B-cell lymphoma 2
4-PBA: 4-phenylbutyric acid
MAPK: Mitogen-activated protein kinase
ERK: Extracellular signal-regulated kinase
SLEDAI: Systemic Lupus Erythematosus Disease Activity Index
L-DMEM: Low-glucose Dulbecco-Modified Eagle Medium
FBS: Fetal bovine serum
PI: Propidium iodide
PBS: Phosphate-buffered saline
PVDF: polyvinylidenedifluoride
SDS-PAGE: SDS-polyacrylamide gel electrophoresis
BH3: BCL-2 homology 3
BIM: BCL-2-interacting mediator of cell death
PUMA: p53 upregulated modulator of apoptosis
BID: BH3-interacting domain death agonist.
